# Structure-Based Understanding of Cu^2+^ Coordination in Fluorescent Proteins for Metal Biosensor Applications—A Review

**DOI:** 10.3390/bios15100675

**Published:** 2025-10-07

**Authors:** Ki Hyun Nam

**Affiliations:** College of General Education, Kookmin University, Seoul 02707, Republic of Korea; structure@kookmin.ac.kr

**Keywords:** fluorescent protein, copper, metal biosensor, structure, interaction

## Abstract

Copper ions play essential roles in biological systems, but they can cause toxicity following dysregulation or excessive accumulation. In addition, environmental overexposure to Cu^2+^ can lead to serious agricultural and ecological issues. Accurate detection of Cu^2+^ is therefore critical in both medical diagnostics and environmental monitoring. Fluorescent proteins (FPs), which are widely used in molecular and cell biology, have been suggested as attractive modalities for metal ion detection owing to their biocompatibility and specific responsiveness to metal ions. The fluorescence emission of FPs is efficiently quenched by Cu^2+^ in a reversible manner, suggesting the potential to develop Cu^2+^-responsive biosensors. To develop highly sensitive and selective Cu^2+^ biosensors based on FPs, an understanding of Cu^2+^ binding to FPs is crucial, along with FP engineering guided by structural analysis. In this study, the molecular properties of FPs and their fluorescence responses to metal ions were reviewed. The crystal structures of FPs complexed with Cu^2+^ were analyzed, revealing both specific and nonspecific Cu^2+^ binding modes. This structural analysis provides insights into the potential of engineering FPs to enhance sensitivity and selectivity for Cu^2+^ detection.

## 1. Introduction

Transition metal ions, such as copper, zinc, and iron, are essential for various biological processes in all living organisms, and play an important role in metabolism [1]. They function as cofactors for enzymes, assist in protein folding and substrate binding, participate in redox reactions, maintain protein structural stability, and regulate cellular signaling [2,3]. Among various transition metal ions, Cu^2+^ serves as a catalytic cofactor in several critical biological functions, such as cytochrome c oxidase for electron transfer during mitochondrial respiration [4], ceruloplasmin for iron transport and antioxidant activity [5], superoxide dismutase 1 for protection against oxidative stress [6], tyrosinase for melanin biosynthesis, and lysyl oxidase for tissue stability [7].

In humans, disrupted copper homeostasis has been implicated in various diseases [8]. For example, Wilson’s disease and Menkes disease are genetic disorders associated with copper imbalance [9,10], and increasing evidence suggests a link between copper accumulation and neurodegenerative diseases, such as Alzheimer’s disease and Parkinson’s disease [11,12]. These findings highlight the importance of the accurate and sensitive detection of copper ions in biological systems.

Beyond biological systems, copper contamination in the environment is a growing concern [13]. The widespread use of copper-based compounds in agriculture, industry, and construction has contributed to the release of copper into water and soil systems [14]. Elevated copper concentrations can disrupt aquatic ecosystems, affect the physiology of aquatic organisms, and alter microbial communities in soil [15].

**Figure 1:**
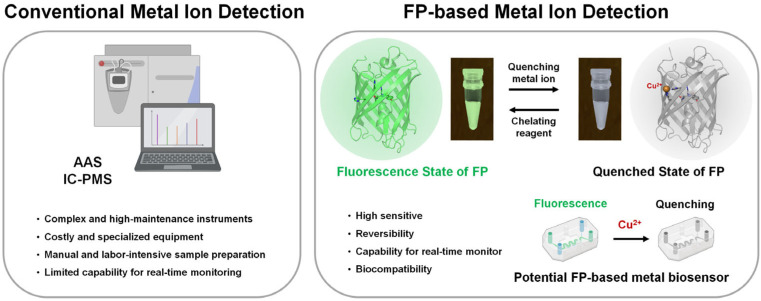
Traditional metal ion analytical techniques and potential fluorescent protein (FP)-based metal ion biosensors. Some images were obtained from BioRender (https://biorender.com/: accessed on 8 March 2025).

Therefore, monitoring copper levels in environmental samples is essential for ecological risk assessment and pollution control [16,17]. Various analytical techniques, such as atomic absorption spectroscopy (AAS) [18], inductively coupled plasma mass spectrometry (ICP-MS) [19], complexonometric assays [20], and electrochemical assays [21], have been developed to detect metal ions, including copper (Figure 1). Among these methods, the limit of detection (LOD) of ICP-MS is typically in the range of a few to several tens of nanomolar [22,23]. In electrochemical assays, the LOD of Cu^2+^ strongly depends on the type of sensor. For example, a Ag nanoparticle-based sensor can detect Cu^2+^ in the range of 1.0–1000 nM with a detection limit of 0.48 nM [24]. A ZnO–graphene photoelectrochemical sensor exhibits a linear detection range from 0.2 µM to 1 mM with a detection limit of 0.03 µM [25]. Using screen-printed electrodes modified with Fe_3_O_4_-embedded carbon nanofibers and multilayer carbon nanotubes, detection limits of 32 nM and 1.04 µM, respectively, have been reported [26]. Accordingly, these methods are highly sensitive and accurate, but they often require sophisticated instruments, expensive equipment, and labor-intensive sample preparation [27]. As such, they are not ideal for real-time monitoring or on-site applications. To measure metal ion levels, various biomolecules such as peptides [28], enzymes [29], antibodies [30], nucleic acids [31], DNAzymes [32], and whole cells [33] have been developed as metal biosensors. However, these biomolecules often face limitations concerning stability, cost, and detection efficiency. In addition, Cu^2+^ detection using organic fluorophores [34], aggregation-induced emission luminogens (AIEgens) [35], and metal–organic frameworks (MOFs) [36,37] has been developed and shown to provide higher sensitivity with lower limits of detection. However, these methods are less suitable for cellular compatibility and are not genetically encodable.

Fluorescence-based detection methods have gained attention because of their high sensitivity, rapid response, and compatibility with in vivo and real-time analysis [38,39,40]. Among fluorescent probes, fluorescent proteins (FPs) are widely used as optical probes in molecular and cell biology studies, as they offer distinct advantages such as a lack of toxicity, the ability to be genetically encoded and expressed in living cells, and the capability for spatiotemporal monitoring of target molecules [41,42]. For efficient FP applications, studies on the discovery of new fluorescent proteins and their engineering are ongoing [43,44]. In addition, FPs have been utilized as versatile biosensors for detecting various factors such as pH and chloride ions [45,46,47,48]. FPs undergo fluorescence quenching in the presence of certain transition metal ions, with Cu^2+^ causing particularly pronounced quenching effects [49,50]. This metal-induced quenching property has attracted considerable interest in the use of FPs as biosensor probes for Cu^2+^ detection (Figure 1). The Cu^2+^-induced fluorescence quenching of FPs has been proposed as a strategy for developing biosensors that respond to changes in copper concentrations [51,52], enable the imaging of copper fluctuations [53], support in vitro diagnostics [51], and permit intracellular copper detection [54]. To develop FPs with high selectivity and sensitivity toward Cu^2+^, protein engineering is essential to minimize the nonspecific binding of other metal ions. For rational protein engineering, a clear understanding of the mechanism by which Cu^2+^ interacts with FPs is crucial, including the identification of potential metal-binding sites. Elucidating the mechanism of Cu^2+^-induced quenching will facilitate the rational design of FP-based biosensors for copper detection in biological or environmental samples. However, the detailed structural basis of Cu^2+^ binding to FPs remains poorly understood.

This review introduces the fluorescence quenching of FPs by transition metal ions. The crystal structures of Cu^2+^-bound FPs were analyzed to identify potential specific and nonspecific Cu^2+^-binding sites. These findings provide insights into the mechanism by which Cu^2+^ interacts with FPs and offer guidance for engineering FPs with improved sensitivity and selectivity for use in Cu^2+^-responsive biosensors.

## 2. Fluorescent Proteins

Green FP (GFP) was first discovered in the jellyfish *Aequorea victoria* [55], and it has subsequently been discovered in other marine species, including corals and sea anemones [56,57]. FPs emit intrinsic fluorescence when excited by specific wavelengths of light. The optical properties of FPs can be affected by the chromophore sequence, surrounding environment, and overall protein conformation [57]. To date, various optical properties of FPs, such as photoactivation, photoswitching, photoconversion, and large Stokes shifts, have been discovered or developed through protein engineering and selectively utilized depending on the research purpose [58,59,60]. In particular, FPs are commonly employed in cellular imaging to monitor gene expression [61], protein localization and trafficking [62], and cellular structures in vivo [63]. Additionally, their ability to participate in fluorescence resonance energy transfer (FRET) enables real-time studies of molecular interactions and conformational changes [64,65].

FPs derived from *A. victoria*, corals, sea anemones, and other species share a highly conserved structure. Generally, FPs consist of 11 β-sheets and two α-helices, forming a single β-barrel fold with a nearly cylindrical shape [56]. The tripeptide corresponding to the chromophore is formed through autocatalytic posttranslational modification processes, including cyclization, dehydration, and oxidation, without the need for external cofactors [57]. The conjugated π-system of the chromophore plays a key role in light absorption and fluorescence emission. The chromophore is located inside the β-barrel, positioned near its center and connected by two α-helices. This rigid β-barrel structure shields the chromophore from water access, thereby enhancing quantum yield and photostability [57]. Mutation of the β-barrel or chromophore region leads to shifts in excitation/emission wavelengths, fluorescence intensity, and photostability [56,57].

The fluorescence intensity of FPs can vary in response to environmental factors such as pH, temperature, and the presence of metal ions [66,67,68]. These environment-sensitive fluorescence changes make FPs useful as biosensors. In particular, pH-dependent fluorescence changes have been widely applied in various imaging experiments [69]. Changes in pH can induce conformational alterations in the FP chromophore [70] or modify the hydrogen-bonding network surrounding the chromophore [71]. Exposure to specific transition metal ions can lead to the quenching of FP fluorescence, suggesting their potential use in the development of metal ion biosensors [68]. To investigate the fluorescence quenching effect of FPs by metal ions, spectroscopic analyses have been conducted for various FPs, including GFP [72], DsRed [51,73,74], Dronpa [75], AmCyan [76], ZsGreen [77], ZsYellow [78], and DendFP [52]. In addition, protein engineering has been employed to enhance metal binding in FP variants, such as BFPms1 [79] and iq-mEmerald [80]. These FPs exhibit significant fluorescence quenching in the presence of metal ions such as Cu^2+^, Fe^2+^, and Fe^3+^. Spectroscopic analyses suggested that that the fluorescence quenching of FPs induced by metal ions can occur through several mechanisms, such as static quenching [81], energy transfer [72], electron transfer [82], and structural perturbation [83].

## 3. Fluorescence Quenching and Reversibility of FP Fluorescence by Cu^2+^

Among various metal ions capable of quenching fluorescence, Cu^2+^ is particularly effective at quenching the fluorescence of various FPs [53,74], highlighting the potential of FPs as sensing probes for Cu^2+^ quantification. For example, the red FP DsRed and its variants (DsRed-Express, DsRed-Monomer, DsRed2, and Rmu13) selectively bind Cu^2+^ even in the presence of other divalent cations, leading to fluorescence quenching [73,74,84]. This quenching effect has been employed to develop in vitro biosensing systems for Cu^2+^ detection by correlating the extent of the fluorescence reduction with the Cu^2+^ concentration [74]. Furthermore, this copper-binding property of DsRed has potential applications in intracellular Cu^2+^ detection through fluorescence-based approaches [51]. Following the initial studies on the potential of DsRed as a Cu^2+^ biosensor, spectroscopic and structural investigations of various FPs in response to metal ions have been conducted to elucidate the mechanisms of fluorescence changes and to discover FPs with enhanced sensitivity to Cu^2+^. To date, various spectroscopic properties of FP in response to Cu^2+^-induced fluorescence quenching have been reported, including quenching efficiency, binding constants, and limits of detection (Table 1).

Spectroscopic analysis of ZsYellow revealed that Cu^2+^ caused the most pronounced quenching effect among the tested metal ions, reducing fluorescence intensity by 81.4% [78]. In addition, Mn^2+^, Co^2+^, Ni^2+^, Zn^2+^, and Cd^2+^ reduced fluorescence by approximately 21.1%, 54.0%, 35.2%, 25.9%, and 32.1%, respectively. These results indicate that ZsYellow exhibits high sensitivity to Cu^2+^ and significant quenching responses to other transition metal ions. Structural analysis further revealed that Cu^2+^ binds nonspecifically to ZsYellow, and the binding affinity is relatively weak.

For AmCyan and mOrange2, Cu^2+^ induced significant quenching, reducing their fluorescence intensities by 90% and 89%, respectively [76]. In addition, AmCyan exhibited quenching in the presence of Co^2+^ and Zn^2+^, with fluorescence reductions of approximately 40% and 50%, respectively, whereas mOrange2 was much less affected by these ions (reduction of approximately 7%). Time-resolved fluorescence measurements after Cu^2+^ addition illustrated that the fluorescence of AmCyan gradually decreased over 5 min, followed by partial recovery, whereas that of mOrange2 steadily declined without recovery throughout the measurement period [76]. The dissociation constant (K_d_) for Cu^2+^ was estimated to be 56.10 µM for AmCyan and 21.46 µM for mOrange2, suggesting differences in Cu^2+^-dependent binding and quenching dynamics between the two FPs.

**Figure 2:**
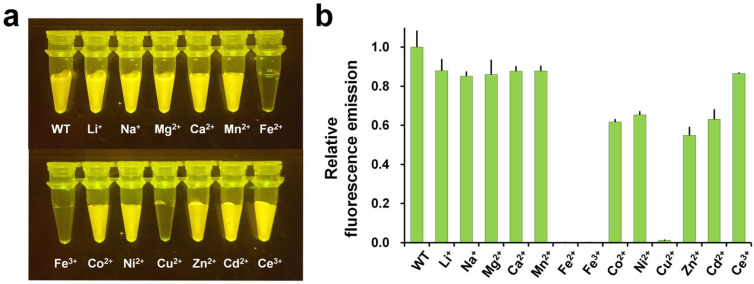
Changes in the fluorescence emission intensity of DendFP (green state) in the presence of metal ions. (**a**) The fluorescence emission of DendFP was significantly quenched by Fe^2+^, Fe^3+^, and Cu^2+^. (**b**) Relative fluorescence emission of DendFP measured in the presence of various metal ions. The original figures were adapted and modified from a previous study [52].

Visible fluorescence quenching was observed for DendFP upon the addition of Fe^2+^, Fe^3+^, and Cu^2+^ (Figure 2a) [52]. Spectroscopic data confirmed that Co^2+^, Ni^2+^, Zn^2+^, and Cd^2+^ also reduced the fluorescence intensity of DendFP (Figure 2b), indicating that this FP is susceptible to fluorescence quenching by several metal ions. Binding affinity analysis revealed that the K_d_ values of Fe^2+^, Fe^3+^, and Cu^2+^ were 24.59, 41.66, and 137.18 µM, respectively, indicating that Fe^2+^ and Fe^3+^ exhibited stronger binding and quenching effects than Cu^2+^.

Collectively, many FPs exhibit high sensitivity to Cu^2+^, highlighting their potential utility as Cu^2+^ biosensor probes. Notably, mOrange2 displayed strong fluorescence quenching, specifically by Cu^2+^, with minimal interference from other metal ions, suggesting favorable selectivity. Contrarily, DendFP exhibited effective quenching by Cu^2+^ but even greater quenching and binding by Fe^2+^ and Fe^3+^, indicating that without protein engineering, DendFP might not be suitable for Cu^2+^-selective sensing applications. Therefore, comprehensive screening against a broad panel of metal ions is essential for assessing the selectivity and suitability of FPs as Cu^2+^ biosensor probes.

To enhance the practical utility of Cu^2+^ biosensors, the FP probe must be reusable. In FP-based Cu^2+^ biosensors, this reusability requires the recovery of fluorescence emission following Cu^2+^ quenching. This reversibility has enabled the development of cost-effective and regenerable FP-based metal ion biosensors. To achieve this, the removal of Cu^2+^ ions bound to FPs is critical. Typically, chelating agents, such as EDTA or EGTA, are used to restore fluorescence by stripping Cu^2+^ from FP. The efficiency of fluorescence recovery depends on the chelating agent concentration. Moreover, the reversibility of fluorescence quenching differs depending on the type of FP used. For example, the fluorescence of AmCyan and mOrange2 was strongly quenched upon Cu^2+^ binding to lower than 20% of their wild-type fluorescence intensity. Treatment with 50 mM EDTA restored their fluorescence intensity to levels exceeding those of the wild-type FPs [76]. In the case of ZsYellow, approximately 90% of the quenched fluorescence was recovered after treatment with 50 mM EDTA [78], whereas DendFP displayed less than 50% recovery under the same conditions [52]. Although the spectroscopic properties of these reversible processes have been extensively studied, the molecular mechanisms underlying the differences in the recovery of FP fluorescence remain unclear. In addition, dialysis has been used to recover fluorescence emission from Cu^2+^-quenched FPs [85]. However, the recovery efficiency by dialysis varies among FPs. For instance, Cu^2+^-quenched mTFP1 recovered approximately 90% of its fluorescence after dialysis, whereas the engineered variant mTFP* exhibited even higher fluorescence recovery [85]. These results suggest that the reversibility efficiency of Cu^2+^-induced fluorescence quenching can be improved through protein engineering.

## 4. Structural Analysis of Cu^2+^ Binding to the FP

To understand the interaction between Cu^2+^ and FP, FP structures deposited in the Protein Data Bank (PDB) were investigated. The search revealed that only four FP structures (mTFP^CHH^, Dronpa, split-GFP, and GHK-GFP) contained Cu^2+^ bound to the protein surface. Accordingly, these FPs were selected for further analysis to provide insights into engineering FPs with improved selectivity and sensitivity for Cu^2+^ detection.

### 4.1. Quenchable Cu^2+^-Bound mTFP^CHH^

Artificial metalloenzymes (ArMs) can be used in biotechnological applications because they integrate the diverse catalytic potential of transition metals with the selective substrate recognition of enzymes [86,87]. Fischer et al. engineered the cyan FP mTFP from *Clavularia* to create a de novo functionalized fluorescent ArM scaffold named mTFP*, resulting in efficient Diels–Alder and Friedel–Crafts alkylase activities [85]. mTFP* was generated by substituting six surface-exposed histidine and methionine residues in mTFP1 (H30Y, M118L, H128Y, H177Y, H178Y, and H209Y) to minimize nonspecific interactions with metal ions. The nonspecific metal binding affinities of mTFP1 and mTFP* were assessed by incubating the proteins with Cu^2+^, Ni^2+^, Rh^3+^, and Pd^2+^ for up to 24 h, followed by attempts to reverse fluorescence quenching through dialysis. For both ArMs, Cu^2+^ induced relatively stronger fluorescence quenching than the other metal ions (Ni^2+^, Rh^3+^, and Pd^2+^). After dialysis, the fluorescence emission of mTFP* incubated with Cu^2+^ was fully recovered, whereas less than 80% recovery was observed for mTFP1. The binding affinity between the FPs and Cu^2+^ was further analyzed using time-resolved metal FRET (tmFRET) titration experiments. Titration fitting of mTFP* yielded Stern–Volmer constants of 0.09 ± 0.01 mM^−1^ at pH 6.0 and 1.12 ± 0.14 mM^−1^ at pH 7.5. Conversely, mTFP1 exhibited at least two distinct K_d_ values to achieve a satisfactory curve fit (R > 0.95): a site-specific K_d_ of 0.02 ± 0.01 μM and a background K_d_ of 330 ± 151 μM at pH 6.0; and values of 2.14 ± 0.29 and 268 ± 125 μM, respectively, at pH 7.5. These results indicate that substituting surface-exposed histidine and methionine residues in the FP can reduce the binding of nonspecific metal ions in mTFP*. However, the binding affinity of metal ions for mTFP* remained significantly low (K_d_ > 1 mM). Fischer et al. further engineered the mTFP* cavity to introduce a specific transition metal ion binding site by substituting I197C–Y200H–Y204H (mTFP^CHH^) and I197E–Y200H–Y204H (mTFP^EHH^) [85]. $K_{d}^{site}$ of Cu^2+^ for mTFP^CHH^ was 0.56 ± 0.06 μM at pH 6.0, versus 0.05 ± 0.01 μM at pH 7.5. For mTFP^EHH^, the corresponding values were 8.2 ± 1.5 and 4.5 ± 2.7 μM, respectively. Liquid chromatography–electrospray ionization mass spectrometry and ICP–MS analyses confirmed the formation of a 1:1 complex between the engineered proteins (mTFP^CHH^ or mTFP^EHH^) and Cu^2+^. By contrast, no Cu^2+^ coordination was detected for mTFP* using these methods.

**Figure 3:**
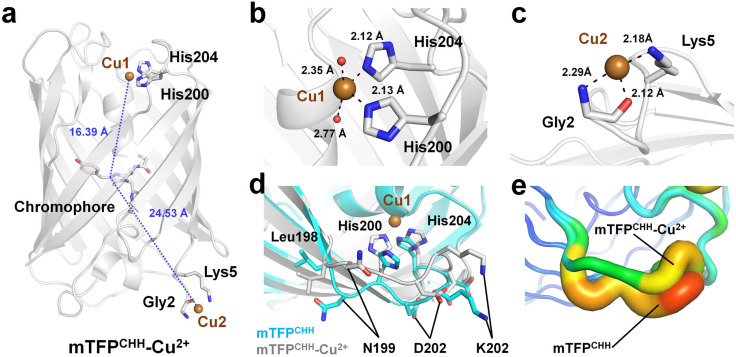
Cu^2+^ binding to mTFP^CHH^ (PDB code: 4R6D). (**a**) Cartoon representation presenting two Cu^2+^ ions bound to mTFP^CHH^. (**b**) Coordination of the Cu^2+^ ion at Cu1, which is located near the α-helix connected to the chromophore of mTFP^CHH^. (**c**) Coordination of the Cu^2+^ ion at Cu2, which is located in the N-terminal region of mTFP*. (**d**) Structural comparison of the Cu1 binding site between Cu^2+^-bound mTFP^CHH^ and apo-mTFP^CHH^ (PDB code: 6QSL). (**e**) B-factor putty representation of the loop between Leu198 and His204 in apo-mTFP^CHH^ and Cu^2+^-bound mTFP^CHH^.

The crystal structure of Cu^2+^-bound mTFP^CHH^ (PDB code: 4R6D) was determined at 1.55 Å resolution. In the deposited Cu^2+^-bound mTFP^CHH^ structure in the PDB, two Cu^2+^ ions were modeled (named Cu1 and Cu2; Figure 3a). Cu1 was located in the cleft of mTFP^CHH^, which is near the α-helix connected to the chromophore of mTFP^CHH^ (Figure 3a). Cu1 was tetragonally coordinated by the NE2 atoms of His200 and His204, along with two water molecules, with coordination distances of 2.13, 2.12, 2.35, and 2.77 Å, respectively (Figure 3b). The distance between Cu1 and the CA2 atom of the chromophore in mTFP^CHH^ was 16.39 Å (Figure 3a). The distance between the chromophore and cleft fell within the appropriate range for tmFRET studies, namely 1.05–2.30 nm [88]. This indicates that metal ions can approach the chromophore within this range and quench its fluorescence via tmFRET [85]. Accordingly, Cu1 located in the engineered region of mTFP^CHH^ represents a quenchable Cu^2+^-binding site.

Cu2 was positioned at the N-terminus (Figure 3a), which was modeled in the deposited PDB but was not discussed in the previous study [85]. Cu2 was coordinated by the backbone nitrogen and oxygen atoms of Gly2 and the NZ atom of Lys5 with coordination distances of 2.29, 2.12, and 2.18 Å, respectively (Figure 3c). The distance between Cu2 and the CA2 atom of the chromophore was 24.5 Å (Figure 3a), suggesting little to no effect on fluorescence quenching, as it lies outside the effective range for tmFRET studies. These findings indicate that Cu^2+^ at the N-terminus can interact nonspecifically with mTFP^CHH^. Although the Cu2 binding site is not directly involved in fluorescence quenching, the N-terminal region modification could be required to avoid nonspecific Cu^2+^ binding and enable precise quantification of Cu^2+^ interactions with mTFP^CHH^.

Accordingly, the Cu1 site is critical for the fluorescence quenching effect in mTFP^CHH^. To understand the mechanism by which Cu1 binding affects the structural changes of mTFP^CHH^, the crystal structure of Cu^2+^-bound mTFP^CHH^ was compared with apo-mTFP^CHH^ (PDB code: 6QSL) determined at 1.6 Å resolution. The superimposition of mTFP^CHH^ and mTFP^CHH^–Cu^2+^ revealed a root mean square deviation of 0.288 Å for all Cα atoms. The superimposed structures revealed that the side chains of His200 and His204 in mTFP^CHH^ shifted toward the inside of the β-barrel cleft upon Cu^2+^ coordination. The distance between the ND1 atom of His204 in apo-mTFP^CHH^ and Cu^2+^-bound mTFP^CHH^ was 1.64 Å, and the distance between the NE2 atom of His200 in the two structures was 3.31 Å (Figure 3d). These positional and conformational changes in His200 and His204 upon Cu^2+^ binding affected the conformation of the loop between Leu198 and His204 (Figure 3d). In addition, Cu^2+^ binding to the cleft of mTFP^CHH^ reduced the flexibility of this loop (Figure 3e). The B-factor values of the loop between Leu198 and His204 from apo-mTFP^CHH^ and Cu^2+^-bound mTFP^CHH^ were 24.73 and 15.32 Å, respectively. These results indicate that Cu^2+^ binding to mTFP^CHH^ affects both the flexibility of the loop regions and the engineered metal-binding site.

### 4.2. Quenchable Cu^2+^-Bound Dronpa

Dronpa is a photoactivatable FP capable of reversible on/off switching between fluorescent and nonfluorescent states using different wavelengths of light [89,90]. This unique property makes this FP highly valuable for superresolution microscopy and dynamic cellular process tracking. Kim et al. performed spectroscopic and structural analyses to investigate whether Dronpa is suitable as a metal biosensor probe [75]. To identify the metal ions that induce fluorescence quenching, purified Dronpa was incubated with various divalent metal ion solutions (Mg^2+^, Ca^2+^, Mn^2+^, Co^2+^, Ni^2+^, Cu^2+^, or Zn^2+^). The fluorescence emission of Dronpa was significantly reduced by 86% in the presence of 50 μM Cu^2+^. Additionally, 50 μM Co^2+^ and Ni^2+^ reduced the fluorescence emission of Dronpa by 26% and 6.3%, respectively. Contrarily, the fluorescence emission of Dronpa was enhanced in the presence of Mg^2+^, Ca^2+^, Mn^2+^, and Zn^2+^. Notably, Cu^2+^-induced fluorescence quenching of Dronpa was highly reversible with approximately 95% recovery.

**Figure 4:**
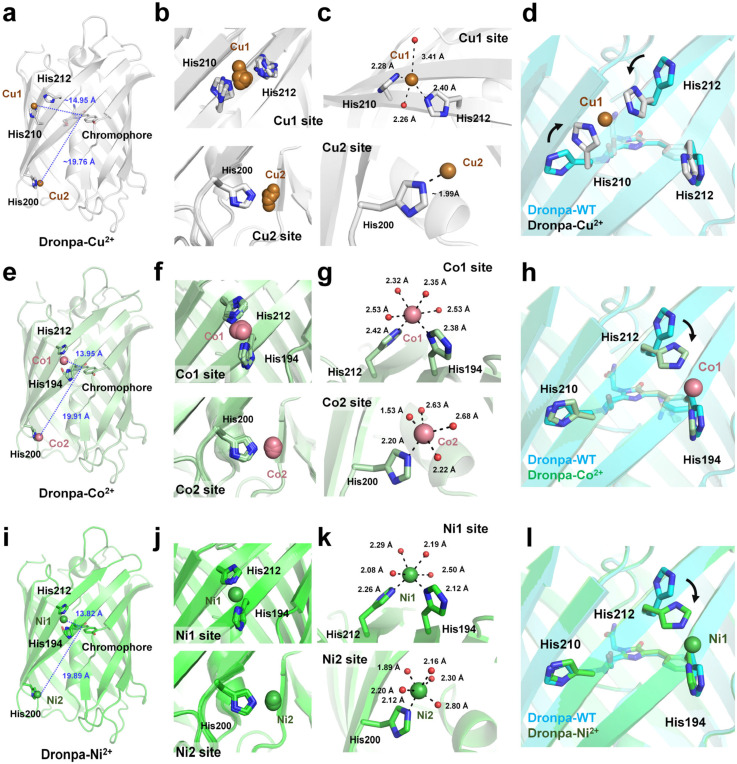
Analysis of metal ion binding to Dronpa. (**a**) Cartoon representation of two Cu^2+^ bound to Dronpa (PDB code: 5HZT). (**b**) Superimposition of the Cu1 and Cu2 binding sites from 24 Dronpa molecules in the asymmetric unit. (**c**) Close-up view of representative Cu1 and Cu2 binding sites in Dronpa. (**d**) Superimposition of the wild-type and Cu^2+^-bound Dronpa structures. (**e**) Cartoon representation of two Co^2+^ bound to Dronpa (PDB code: 5HZS). (**f**) Superimposition of the Co1 and Co2 binding sites from 24 Dronpa molecules in the asymmetric unit. (**g**) Close-up view of representative Co1 and Co2 binding sites in Dronpa. (**h**) Superimposition of the wild-type and Co^2+^-bound Dronpa structures. (**i**) Cartoon representation of two Ni^2+^ bound to Dronpa (PDB code: 5HZU). (**j**) Superimposition of the Ni1 and Ni2 binding sites from 24 Dronpa molecules in the asymmetric unit. (**k**) Close-up view of representative Ni1 and Ni2 binding sites in Dronpa. (**l**) Superimposition of the wild-type and Ni^2+^-bound Dronpa structures. The conformational change in the histidine side chain upon metal ion binding is indicated by an arrow.

To determine the quenchable metal ion binding site, the crystal structure of Dronpa soaked with Cu^2+^ was determined at 2.84 Å resolution (PDB code: 5HZT). The crystal belongs to the triclinic P1 space group and contains 24 molecules in the asymmetric unit. Two Cu^2+^ ions (designated Cu1 and Cu2) were bound to the Dronpa molecule (Figure 4a). Cu1 was located on the surface at the midpoint of the β-barrel fold, positioned perpendicular to the Dronpa chromophore. Superimposition of the 24 Dronpa molecules revealed slight positional variation of the Cu1 and C2 sites (Figure 4b). The Cu1 ion was commonly coordinated by His210 and His212, and the side chains of two Lys37 residues from nearby Dronpa molecules also interacted with Cu1. However, the side-chain conformations of the metal-interacting histidine residues were not identical, indicating that Cu1 is not tightly bound (Figure 4b). Among all Cu1 sites, the electron density map for chain H was relatively clearer than in the other Cu^2+^-bound Dronpa molecules. In chain H, the Cu1 ion was distorted tetrahedrally coordinated by the NE2 atoms of His210 and His212 and two water molecules at distances of 2.28, 2.40, 2.26, and 3.41 Å, respectively (Figure 4c). For the 24 molecules in the asymmetric unit, the distances between Cu1 and the NE2 atoms of His210 and His212 in the 24 Dronpa molecules ranged from 2.10 to 2.95 Å and 2.11 to 3.01 Å, respectively. The distance between Cu1 and the CA2 atom of the chromophore ranged from 14.62 to 15.29 Å, and the shortest distance between Cu1 and the tyrosine ring of the Dronpa chromophore ranged from 12.67 to 13.68 Å. These values suggest that the Cu1 site, coordinated by His210 and His212, is responsible for quenching, as supported by previous FRET studies [88].

A second Cu^2+^ site (Cu2) was located at the bottom of the β-barrel. The Cu^2+^-binding site was not analyzed and described in the original manuscript [75]. Cu2 was observed in all Dronpa molecules in the asymmetric unit and was coordinated by the NE2 atom of His220, located at the edge of the β-strand (Figure 4c). Superimposition of the 24 Dronpa molecules illustrated that the side-chain conformation of His220 was conserved, although the position of Cu2 varied slightly, indicating weak coordination. The distance between Cu2 and the CA2 atom of the chromophore ranged from 19.27 to 20.26 Å (Figure 4a). Although this Cu^2+^-binding site has not been previously studied in detail, the distance suggests that Cu2 also contributes to fluorescence quenching, which warrants further investigation.

To understand Cu^2+^-induced structural changes at the binding site, the side-chain conformations of His210 and His212 were compared between the Cu^2+^-bound and native Dronpa structures. Superimposition demonstrated that the His210 and His212 side chains point away from the Cu^2+^-binding site in the native Dronpa structure. The average rotation angles of the His210 and His212 side chains were approximately 90 and 115°, respectively (Figure 4d). These findings suggest that Cu^2+^ binding can induce conformational changes in the metal-coordinating residues.

Compared with the findings for wild-type Dronpa, Co^2+^ and Ni^2+^ ions also reduced the fluorescence emission of Dronpa by 26% and 6.3%, respectively. The crystal structures of Co^2+^- and Ni^2+^-bound Dronpa were determined at a resolution of 1.90 and 2.15 Å, respectively. Two Co^2+^ ions and two Ni^2+^ ions were bound at two nearly identical sites on the FP surface, respectively (Figure 4e,i). Superimposition of the 24 molecules in the asymmetric unit of the Dronpa–Co and Dronpa–Ni structures revealed slight variations in metal ion positions, suggesting that Co^2+^ and Ni^2+^ are not rigidly bound (Figure 4f,j). The first metal binding site (Co1/Ni1) was octahedrally coordinated by His194, His212, and water molecules (Figure 4g,k). However, the coordination angles and distances slightly differed [75]. The second metal binding site (Co2/Ni2) was coordinated by His200 and was located near the N-terminal α-helix of the β-barrel (Figure 4g,k). Superimposition of the 24 molecules in the asymmetric unit of the Dronpa–Co and Dronpa–Ni structures revealed slight variations in the positions of the metal ions. The distances of Co1/Ni1 and Co2/Ni2 to the CA2 atom of the Dronpa chromophore were approximately 13.75–14.15/13.76–13.89 and 19.67–20.15/19.72–20.06 Å, respectively. These results suggest that Co1 are more likely responsible for the observed fluorescence reduction for Co^2+^. Compared with the findings for the native structure, the side chains of His194 and His212 exhibited conformational changes upon Co^2+^ or Ni^2+^ binding (Figure 4h,l), whereas no such changes were noted for His210 side chains. These findings indicate that although the Co^2+^ and Ni^2+^ binding sites and coordination geometries are similar, their fluorescence quenching effects differ significantly, underscoring the importance of the specific metal ion involved.

The positions and coordination geometries of Cu1 versus Co1/Ni1 were notably different even though all three ions were coordinated by His212, suggesting that the preferred binding position depends on the identity of the coordinating histidine residues [75]. By contrast, Cu2, Co2, and Ni2 were all coordinated by His200 alone, indicating a lack of binding selectivity at this site.

### 4.3. Cu^2+^ Bound to the Oligomeric Interface of Split-GFP

The spatial organization of multiple proteins has potential applications in synthetic biology, including metabolic pathway optimization [91], spatial arrangement of signaling molecules [92], and self-assembling protein architecture construction [93]. The development of efficient systems for precise protein spatial organization could also significantly advance research in protein crystallization [94]. Leibly et al. selected split-GFP, consisting of a GFP core (strands 1–9) and a GFP hairpin (strands 10–11), as a scaffold and engineered 11 distinct oligomeric forms by introducing specific disulfide bonds or metal-binding sites [95]. They explored the potential of forming GFP dimers and higher-order oligomers by designing metal-binding half-sites on the GFP surface, with metal ions such as Cu^2+^, Zn^2+^, or Ni^2+^ playing a stabilizing role by bridging multiple GFP units. Ultimately, this study did not focus on the fluorescence quenching of FPs by metal ion; therefore, no spectroscopic analysis was performed to assess the fluorescence quenching effect of metal ion bridging on GFP. However, the structural characterization of Cu^2+^ binding to FPs could provide valuable insights for the development of FP-based Cu^2+^ biosensors. The detailed structural properties of Cu^2+^ binding at the interface of the GFP oligomer were analyzed in this study.

In this study, 33 crystal structures of oligomeric states of split-GFP were determined, and they can be broadly categorized into four types: disulfide-mediated, metal ion-mediated, disulfide and metal contacts, and Cu^2+^ dimers. Only the five structures (PDB code: 4W6T, 4W7D, 4W7E, 4W7F, and 4W7R) that exclusively exhibited Cu^2+^-mediated oligomerization, excluding those involving both disulfide and Cu^2+^ interactions, were analyzed in this study.

**Figure 5:**
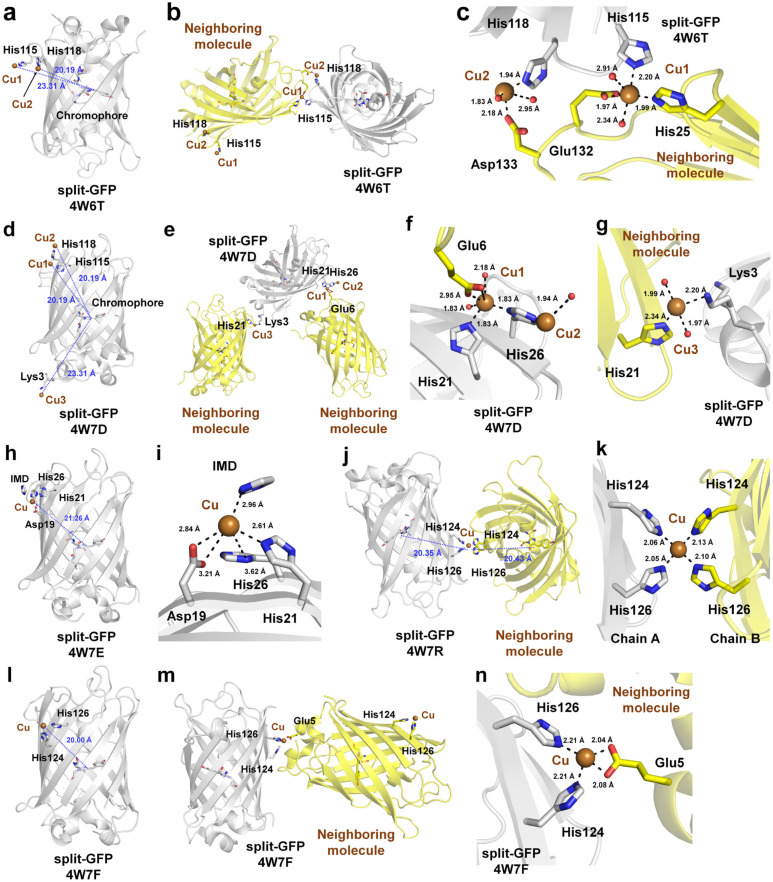
Crystal structure of Cu^2+^-bound split-GFP. (**a**) Cartoon representation of Cu^2+^-bound split-GFP (PDB code: 4W6T). (**b**) Cu^2+^-mediated interaction between the split-GFP molecules (PDB code: 4W6T) within the crystal lattice. (**c**) Close-up view of the Cu^2+^-mediated interface between split-GFPs (PDB code: 4W6T). (**d**) Cartoon representation of Cu^2+^-bound split-GFP (PDB code: 4W7D). (**e**) Cu^2+^-mediated interaction between split-GFP molecules within the crystal lattice (PDB code: 4W7D). Close-up views of (**f**) the Cu1 and Cu2 sites and (**g**) the Cu3 site revealing the Cu^2+^-mediated interfaces between split-GFPs (PDB code: 4W7D). (**h**) Cartoon representation of Cu^2+^-bound split-GFP (PDB code: 4W7E). (**i**) Close-up view of the Cu^2+^-binding site (PDB code: 4W7E). The Cu^2+^ ion did not interact with the neighboring molecules. IMD, imidazole. (**j**) Cu^2+^-mediated interaction between split-GFP molecules within the crystal lattice (PDB code: 4W7R). (**k**) Close-up view of the Cu^2+^-binding site (PDB code: 4W7R). (**l**) Cartoon representation of Cu^2+^-bound split-GFP (PDB code: 4W7F). (**m**) Cu^2+^-mediated interaction between split-GFP molecules within the crystal lattice (PDB code: 4W7F). (**n**) Close-up view of the Cu^2+^-binding site (PDB code: 4W7F).

For split-GFP-4W6T, two Cu^2+^ ions bound to the NE2 atoms of the engineered residues E115H and T118H at distances of 2.20 and 1.94 Å, respectively (Figure 5a). These two Cu^2+^ ions facilitated intermolecular interactions between adjacent split-GFP molecules within the crystal lattice (Figure 5b). The Cu^2+^ bound to E115H is also coordinated by the NE2 atom of His25, the OE1 and OE2 atoms of Glu132 from neighboring molecules in the crystal lattice, and two water molecules at distances of 1.99, 1.97, 2.93, 2.34, and 2.91 Å, respectively (Figure 5c). The Cu^2+^ bound to T118H was additionally coordinated by the OD2 atom of Asp133 from neighboring molecules in the crystal lattice and two water molecules at distances of 2.18, 1.83, and 2.95 Å, respectively (Figure 5c). The distances from the Cu^2+^ ions interacting with E115H and T118H to the CA2 atom of the split-GFP chromophore were 23.31 and 20.19 Å, respectively.

For split-GFP-4W7D, two split-GFP molecules were present in the asymmetric unit, each exhibiting distinct Cu^2+^ binding patterns. In chain A, three Cu^2+^ ions were modeled (Figure 5d), whereas no Cu^2+^ ions were modeled in chain B. However, in chain B, an interaction between a Cu^2+^ ion and a symmetry-related molecule was observed (Figure 5e). The distances from Cu1, Cu2, and Cu3 to the CA2 atom of the chromophore were 22.87, 26.98, and 28.81 Å, respectively (Figure 5d). In chain A, the first Cu^2+^ (Cu1) was coordinated by the NE2 atom of His21, the ND1 atom of His26, the OE2 atom of Glu6 from a neighboring molecule in the crystal lattice, and two water molecules with coordination distances of 2.27, 2.04, 2.06, 2.65, and 2.86 Å, respectively (Figure 5f). The second Cu^2+^ ion (Cu2) interacted with the NE2 atom of His26 and one water molecule at distances of 1.97 and 2.82 Å, respectively (Figure 5f). The third Cu^2+^ ion (Cu3) was coordinated by the NZ atom of Lys3, the NE2 atom of His21 from a neighboring molecule, and two water molecules at distances of 2.50, 2.06, 2.55, and 2.65 Å, respectively (Figure 5g). For split-GFP-4W7E, one Cu^2+^ was bound to the FP surface (Figure 5h). The distance between Cu^2+^ and the CA2 atom of the chromophore was 21.26 Å. Cu^2+^ was coordinated by the OD1 and OD2 atoms of Asp19, NE2 atom of His21, and N1 atom of an imidazole molecule derived from the crystallization solution (Figure 5i). The coordination distances were 3.21, 2.84, 2.61, and 2.96 Å, respectively. Cu^2+^ was additionally located near a neighboring molecule, but it did not interact with any neighboring residues in the crystal lattice (Figure 5i). Four FP molecules were present in the asymmetric unit of split-GFP-4W7R, and two Cu^2+^ molecules were located at the interface between two split-GFP molecules, exhibiting identical coordination patterns (Figure 5j). The distances between the Cu^2+^ ions and the CA2 atoms of the chromophores from the four split-GFP molecules ranged from 20.35 to 20.43 Å (Figure 5j). Each Cu^2+^ ion was coordinated by the NE2 atoms of His124 and His126 from two separate split-GFP molecules, with distances of 2.04–2.13 Å and 2.05–2.10 Å, respectively, in the four molecules of the asymmetric unit (Figure 5k). For split-GFP-4W7F, one Cu^2+^ ion was bound to the FP surface (Figure 5l). The distance between Cu^2+^ and the CA2 atom of the chromophore was 20.00 Å. The Cu^2+^ ion mediated an interaction with a neighboring molecule (Figure 5m). This ion was coordinated by the NE2 atoms of His124 and His126, both at distances of 2.21 Å. Additionally, this Cu^2+^ interacted with the OE1 and OE2 atoms of Glu5 from a neighboring molecule in the crystal lattice with coordination distances of 2.04 and 2.08 Å, respectively (Figure 5n).

Taken together, the split-GFP structures demonstrated that even for the same protein, Cu^2+^ can bind at various positions, exhibiting distinct binding modes through interactions with different combinations of amino acid residues. From the perspective of Cu^2+^ biosensor probe development, these results suggest that Cu^2+^ can form stable yet nonspecific interactions with various amino acid residues, particularly histidine, on the surface of FPs.

### 4.4. Cu^2+^-Binding GHK Tripeptide Fused to GFP

The phase problem remains a major bottleneck in protein crystallography because X-ray diffraction experiments only provide the amplitudes of scattered X-rays but not their phases [96]. Various experimental phasing methods have been developed to overcome this limitation, including molecular replacement, single- and multi-wavelength anomalous dispersion, and single- and multiple isomorphous replacement (SIR and MIR) [96].

Mehr et al. reported the high-affinity Cu^2+^-binding tripeptide GHK genetically fused to the N-terminus of a GFP variant and the fused peptide MBP–FG [97]. This study demonstrated that incorporation of the GHK–Cu^2+^ complex both facilitated crystallization and enabled successful experimental phasing using copper single-wavelength anomalous dispersion. Because the purpose of their study was unrelated to the effect of Cu^2+^ on fluorescence, it did not investigate the influence of Cu^2+^ binding to the GHK–GFP variant on the fluorescence intensity of the FP. Nevertheless, the interaction of the Cu^2+^ ion bound to the GHK tag at the N-terminus of GFP could offer structural insights for the future development of Cu^2+^ biosensors. Therefore, in the present study, the coordination of Cu^2+^ ions within the crystal structure of GHK-tagged GFP was analyzed in greater detail than in the previous report.

**Figure 6:**
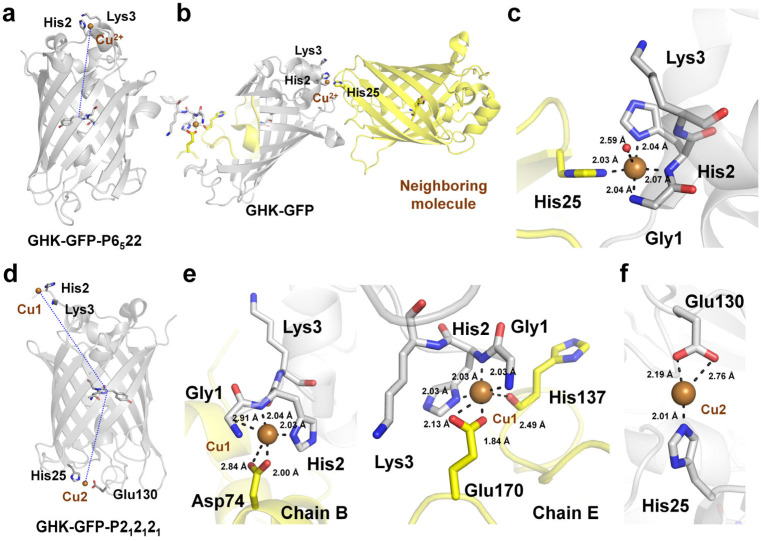
Crystal structure of Cu^2+^ bound to the GHK tripeptide fused at the N-terminus of GFP. (**a**) Cu^2+^ binding to the GHK tripeptide in the GFP structure with the P6_5_22 crystal form (PDB code: 6QUJ). (**b**) Interaction of Cu^2+^ bound to GHK-GFP with the neighboring molecule. (**c**) Close-up view of the Cu^2+^ coordination in GHK-GFP-P6_5_22, including interaction with the neighboring molecule. (**d**) Cu^2+^ binding to the GHK tripeptide in the GFP structure with the P2_1_2_1_2_1_ crystal form (PDB code: 6QUH). (**e**) Close-up view of the Cu^2+^ coordination in GHK-GFP-P2_1_2_1_2_1_, including interaction with the neighboring molecule. (**f**) Close-up view of the Cu2 coordination in GHK-GFP-P2_1_2_1_2_1_.

The crystal structures of GHK–GFP complexed with Cu^2+^ were determined in two different crystal forms: P6_5_22 (PDB codes: 6QUJ and 6QUI) and P2_1_2_1_2_1_ (PDB code: 6QUH). The two GHK–GFP structures in the P6_5_22 crystal form contained two molecules in the asymmetric unit, each exhibited a Cu^2+^ ion bound to the GHK tripeptide at the GFP N-terminus (Figure 6a). The Cu^2+^ ion binds to the GHK peptide of GFP and also interacts with neighboring GFP molecules in the crystal lattice (Figure 6b). For GHK-GFP-6QUJ, Cu^2+^ ion was coordinated by the N atom of glycine, N atom of histidine, and ND1 atom of histidine from the GHK tripeptide in all molecules of the P6_5_22 form, and bond distances of 2.04/2.06 Å, 2.07/2.06 Å, and 2.07/2.03 Å for chain A/B, respectively (Figure 6c). In addition, Cu^2+^ was coordinated by the NE2 atom of His25 from a neighboring molecule in the crystal lattice and a water molecule at distances of 1.98–2.19 and 2.38–2.60 Å, respectively (Figure 6c). Although the exact coordination distances varied slightly, the square–pyramidal coordination geometry of the Cu^2+^ ion by the GHK tripeptide, His25, and a water molecule was essentially identical in all four GHK–GFP structures. The Cu^2+^ binding in GHK-GFP-6QUI were similar with GHK-GFP-6QUJ.

In the P2_1_2_1_2_1_ crystal form, the structure contained two molecules in the asymmetric unit, each with one Cu^2+^ ion bound to the GHK–GFP molecule (Figure 6d). In chains B/E (based on PDB assignment), Cu^2+^ was coordinated by the N atom of glycine, N atom of histidine, and ND1 atom of histidine from the GHK tripeptide at distances of 2.03/2.03, 2.04/2.03, and 2.03/2.03 Å, respectively (Figure 6e). The Cu^2+^ ion was also coordinated by residues from neighboring molecules, although the specific interactions differed because of variations in crystal packing, likely attributable to the different conformations of the Cu^2+^-bound GHK. In chain B, the GHK-bound Cu^2+^ was further coordinated by the OD1 and OD2 atoms of Asp74 from a neighboring molecule at distances of 2.00 Å and 2.84 Å, respectively (Figure 6e). In chain E, the Cu^2+^ is coordinated by the OE1 and OE2 atoms of Glu170 from a neighboring molecule, the main-chain carbonyl group of His137, and a water molecule at distances of 3.13 Å, 1.84 Å, and 2.49 Å, respectively (Figure 6e).

A second Cu^2+^-binding site was observed on the opposite side of the GHK tripeptide region on GFP. In chain B, this solvent-exposed second Cu^2+^ molecule had an occupancy of 0.29, and it was coordinated by the NE2 atom of His25, the OE1/OE2 atoms of Glu130, and a water molecule at distances of 2.03 2.12/2.13, and 2.41 Å, respectively (Figure 6d). In chain E, the second Cu^2+^ site had full occupancy (1.00), and it was coordinated by the NE2 atom of His25, and the OE1/OE2 atoms of Glu130 at distances of 2.01, and 2.19/2.76 Å, respectively (Figure 6f). Accordingly, the occupancy and coordination of the second Cu^2+^ site differed significantly depending on the crystal packing.

## 5. Discussion

FPs are widely used to monitor molecular functions in molecular and cell biology, and they also serve as biosensors that respond to environmental changes. In particular, metal ion-induced fluorescence quenching represents a useful strategy for detecting specific metal ions, such as Cu^2+^, with high sensitivity and in real time. Numerous studies have focused on the application of FPs as biosensors for detecting metal ions. Among the various metal ions that induce quenching, this review focused on Cu^2+^-induced fluorescence quenching and the associated metal-binding characteristics of FPs. Spectroscopic analyses have revealed that many FPs commonly exhibit high sensitivity to Cu^2+^-induced quenching, although their responses to other metal ions vary.

In real-world samples, multiple metal ions coexist with Cu^2+^, making selective quenching by Cu^2+^ a key requirement for biosensor development. For this reason, FPs that exhibit highly sensitive and selective quenching by Cu^2+^, such as mOrange2, are promising biosensor candidates. Conversely, FPs such as DendFP and ZsGreen exhibit stronger quenching in the presence of Fe^2+^ and Fe^3+^ than in the presence of Cu^2+^. Although these FPs might be suitable for detecting iron, their susceptibility to Cu^2+^ interference limits their utility as selective Fe ion sensors. Similarly, FPs such as ZsYellow and Dronpa also exhibit reduced fluorescence in the presence of Co^2+^ and Zn^2+^, which could lead to inaccurate Cu^2+^ quantification. Therefore, the discovery of novel FPs that selectively respond to Cu^2+^ or the engineering of proteins to bind Cu^2+^ specifically is critical.

The fluorescence recovery of Cu^2+^-quenched FPs by chelating reagents or dialysis has been reported, providing preliminary insights into the potential reusability of FP-based biosensors. However, the speed and reliability of this reversibility have not been thoroughly evaluated for real-time or continuous monitoring applications. From an applied perspective, further in-depth studies are required to establish efficient and rapid recovery processes to enable continuous Cu^2+^ sensing.

**Figure 7:**
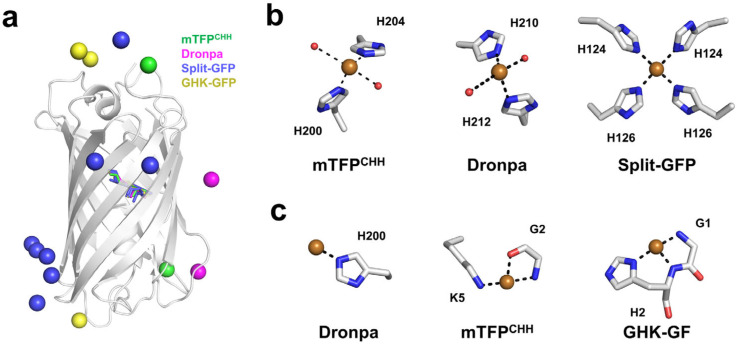
Visual schematic summarizing Cu^2+^ binding in fluorescent proteins (FPs). (**a**) Superimposition of Cu^2+^ binding sites in mTFP^CHH^, Dronpa, split-GFP, and GHK-GFP. The bound Cu^2+^ ions in mTFP^CHH^, Dronpa, split-GFP, and GHK-GFP are shown as green, pink, blue, and yellow spheres, respectively. (**b**) Representative tetrahedral or square planar coordination geometries of Cu^2+^ observed in FPs. (**c**) Example of an unidentified Cu^2+^ coordination geometry in FPs.

In this study, four different crystal structures of Cu^2+^-bound FPs were analyzed. Cu^2+^ was found to bind not only near the chromophore but also at the N-terminal or flexible regions of the proteins (Figure 7a). Cu^2+^ interacted with the imidazole ring of histidine, the carboxyl groups of aspartate or glutamate residues, or nonspecifically with the N-terminal backbone. Cu^2+^ generally prefers 4–6 coordination [98,99]. Meanwhile, the Cu^2+^ bound to FPs exhibited diverse coordination geometries. For example, in mTFP^CHH^ and Dronpa, Cu^2+^ displayed a tetrahedral coordination involving histidine residues and water molecules (Figure 7b). In split-GFP (PDB: 4W7R), Cu^2+^ was coordinated in a square planar geometry by four histidine residues (Figure 7b). In contrast to such stable coordination geometries, in some cases, such as in Dronpa, Cu^2+^ interacted with only a single histidine residue, or with main-chain atoms, making it difficult to clearly define a specific coordination geometry (Figure 7c). Overall, Cu^2+^ can bind to multiple regions of FPs and adopt diverse coordination geometries through interactions with amino acid side chains and water molecules. Therefore, precise identification of Cu^2+^ binding sites and coordination modes requires experimental structural determination.

Structural analysis of Cu^2+^-bound FPs showed that Cu^2+^ commonly interacts with surface-exposed histidine or aspartic acid residues, but the binding specificity varies among FPs. For example, in mTFP^CHH^ and Dronpa, despite the presence of multiple histidine and aspartic acid residues on the FP surface, Cu^2+^ binds at specific residues. In contrast, in split-GFP and GHK-GFP, Cu^2+^ interacts with different histidine or aspartic acid residues in a more nonspecific manner. Collectively, these results indicate that the binding specificity of Cu^2+^ differs among FPs. In the case of mTFP*, engineering efforts involved substituting surface histidine and methionine residues to prevent nonspecific metal binding [85]. Titration experiments confirmed the reduction in nonspecific interactions and illustrated that fluorescence was effectively recovered after dialysis. This strategy highlights the effectiveness of removing potential metal-binding residues to improve FP recovery and reduce background interference, ultimately enhancing the accuracy of metal quantification. Regarding Dronpa, the Cu^2+^-binding sites were distinct from those of Co^2+^ and Ni^2+^ [75], suggesting that selective Cu^2+^ quenching can be achieved by mutating Co^2+^- or Ni^2+^-binding residues while preserving Cu^2+^-binding ability. In split-GFP, Cu^2+^ bound at multiple locations within the same protein, potentially inducing protein aggregation [95]. Cu^2+^ tends to coordinate with surface histidine residues, thereby promoting interactions between neighboring FPs and leading to aggregation. Such aggregation might hinder accurate Cu^2+^ quantification, indicating the need to replace surface residues to prevent nonspecific Cu^2+^ binding for metal biosensor applications. Concerning GFP fused with the GHK tripeptide, the design was intended for specific Cu^2+^ binding [97]. This result illustrated that the presence of potential metal ion binding sites, such as the N-terminal backbone nitrogen, could permit nonspecific Cu^2+^ interactions. Although the chromophore is distant from this site and quenching might not occur, nonspecific binding could lead to inaccurate Cu^2+^ quantification. Thus, truncating the N- or C-terminal regions or substituting metal-preferred residues in FP-based probes could help eliminate nonspecific interactions. Structural data illustrated that Cu^2+^ binds to histidine and aspartate residues on the FP surface. To eliminate nonspecific Cu^2+^ interactions, surface histidine residues not involved in targeted sensing should be replaced.

To identify the quenchable Cu^2+^-binding site on FPs, experimental approaches such as co-crystallization of FPs with Cu^2+^ or soaking FP crystals into the crystallization solutions containing Cu^2+^ are commonly employed. These methods have been successfully used to obtain the crystal structures of Cu^2+^-bound Dronpa, mTFP, split-GFP, and GHK–GFP. However, co-crystallization and soaking with quenchable metal ions were unsuccessful in the case of DendFP because of protein precipitation or crystal damage. This suggests that DendFP has higher sensitivity to structural perturbation by quenchable Cu^2+^ or that its surface residues involved in quenchable Cu^2+^ binding cause aggregation or interfere with crystal packing. Given these challenges, a possible approach for future studies would be to start from extremely low concentrations and gradually increase the concentration of quenchable Cu^2+^ during the soaking process. This stepwise method could help minimize crystal damage and facilitate the identification of quenchable Cu^2+^-binding sites in FP.

Meanwhile, in the crystallographic study of ZsYellow with quenchable Cu^2+^, the color of ZsYellow crystals successfully changed to a quenched state after soaking in a crystallization solution containing Cu^2+^. However, Cu^2+^ was not observed in the electron density map, suggesting that Cu^2+^ bound nonspecifically to ZsYellow. This result is consistent with that of spectroscopic analysis, which indicated a low binding affinity of Cu^2+^ for ZsYellow. These findings suggest that although Cu^2+^ can reduce or quench the fluorescence emission of FPs, this might not be observed in the crystal structure if the binding affinity between the protein and Cu^2+^ is low because of weak or nonspecific interactions. Therefore, to identify Cu^2+^-binding sites in FPs, it is important to first evaluate the binding affinity between the FP and Cu^2+^, which can help assess the feasibility of crystallographic experiments.

When structural information is not available, nonspecific Cu^2+^ binding could be reduced by substituting surface-exposed histidine and aspartic acid residues on the β-barrel fold with other amino acids. However, such mutations may alter the fluorescence properties of the FP. Therefore, accurate identification of nonspecific Cu^2+^ binding sites and targeted substitution of the involved residues essentially require experimentally determined Cu^2+^-bound FP structures. When experimental structures are not available, metal ion docking to FPs can serve as an alternative approach [100]. Recent studies have shown that, although such models do not provide precise coordination geometries, some Cu^2+^ docking models can reliably predict potential binding sites when compared with experimentally determined Cu^2+^-bound FP structures [100].

Overall, FP-based Cu^2+^ biosensors are still at the developmental stage; nevertheless, they hold promise for diverse future applications, including in vivo imaging platforms for biomedical diagnostics, as well as environmental and food monitoring.

## Figures and Tables

**Table 1:** Comparison of the Cu^2+^-induced fluorescence quenching properties of FPs.

FP	Quenching Efficiency (%)	*K*_d_ (μM)	Recovery (EDTA)	Limit of Detection (LOD)	Reference
DsRed	90.0 ± 10	0.54	ND	45 ± 2 nM	[74]
drFP583	78.0	14.80 ± 1.68	>90% (1 mM)	ND	[73]
Rmu13	66.0	10.90 ± 1.74	ND	ND	[73]
Dronpa	86.0	ND	ND	ND	[75]
AmCyan	80.0	56.10	89.4% (5 mM)	ND	[76]
mOrange2	89.0	21.46	>100% (5 mM)	ND	[76]
ZsYellow	81.4	ND	ND	ND	[78]
ZsGreen	77.2	68.2	>90% (50 mM)	ND	[77]
DendFP	98.8	137.18	44.2% (50 mM)	3.2 μM	[52]

ND: not discussed.

## Data Availability

No new data were created or analyzed in this study.
